# RVCNet: A hybrid deep neural network framework for the diagnosis of lung diseases

**DOI:** 10.1371/journal.pone.0293125

**Published:** 2023-12-28

**Authors:** Fatema Binte Alam, Prajoy Podder, M. Rubaiyat Hossain Mondal

**Affiliations:** Institute of Information and Communication Technology, Bangladesh University of Engineering and Technology, Dhaka, Bangladesh; PLOS, UNITED KINGDOM

## Abstract

Early evaluation and diagnosis can significantly reduce the life-threatening nature of lung diseases. Computer-aided diagnostic systems (CADs) can help radiologists make more precise diagnoses and reduce misinterpretations in lung disease diagnosis. Existing literature indicates that more research is needed to correctly classify lung diseases in the presence of multiple classes for different radiographic imaging datasets. As a result, this paper proposes RVCNet, a hybrid deep neural network framework for predicting lung diseases from an X-ray dataset of multiple classes. This framework is developed based on the ideas of three deep learning techniques: ResNet101V2, VGG19, and a basic CNN model. In the feature extraction phase of this new hybrid architecture, hyperparameter fine-tuning is used. Additional layers, such as batch normalization, dropout, and a few dense layers, are applied in the classification phase. The proposed method is applied to a dataset of COVID-19, non-COVID lung infections, viral pneumonia, and normal patients’ X-ray images. The experiments take into account 2262 training and 252 testing images. Results show that with the Nadam optimizer, the proposed algorithm has an overall classification accuracy, AUC, precision, recall, and F1-score of 91.27%, 92.31%, 90.48%, 98.30%, and 94.23%, respectively. Finally, these results are compared with some recent deep-learning models. For this four-class dataset, the proposed RVCNet has a classification accuracy of 91.27%, which is better than ResNet101V2, VGG19, VGG19 over CNN, and other stand-alone models. Finally, the application of the GRAD-CAM approach clearly interprets the classification of images by the RVCNet framework.

## 1. Introduction

Lung disorders affect millions of people globally and encompass a range of conditions that impede proper lung function. Viral or bacterial infections cause different types of lung problems. Environmental factors, such as air pollution and smoking, can cause clinical disorders such as novel coronavirus disease (COVID-19), non-COVID lung infections, viral pneumonia, asthma, lung cancer, and tuberculosis [1]. According to the Forum of the International Respiratory Society (FIRS), asthma affects nearly 350 million individuals annually, and 4 million die from lung infections and pneumonia [2]. The success of artificial intelligence (AI) models in various domains has motivated the development of AI models for medical image analysis for detecting various virus-related diseases, including lung disorders [9, 10]. Early detection and diagnosis are crucial for the effective treatment of these diseases. While traditional methods have been utilized to diagnose lung ailments, they lack sufficient respiratory experts and medical equipment [3, 4].

Recent advancements in machine learning (ML) and deep learning (DL) have led to increased utilization of medical image analysis, particularly for automating the process and reducing operator variability [5–15]. However, for reading medical images, professionals must integrate intuitive analysis results with regulated diagnostic procedures. Traditionally, computers execute specialized algorithms to solve only rule-based problems. This method cannot calculate and implement all necessary medical image analysis-based diagnosis steps. There is a need for algorithms that mimic human intuition to enhance medical image analysis, and this is where AI comes in. AI simulates human instinct on computers, with training being the most crucial component of algorithms that mimic human intuition. DL techniques have been employed for other disease detections, such as Chickenpox, Measles, Herpes Zoster Virus (HZV), and Ebola virus disease, with promising results in accuracy and performance [14, 16–18]. This highlights the potential of using transfer learning and DL in the context of lung disorder detection.

Like the human visual system, computer algorithms can modify internal parameters and structures during training. This limited enrollment capacity suffices since the rule-based diagnosis method offers the necessary focus and clarity to fix the problem intuitively. However, traditional AI algorithms have limitations since medical image processing problems require unique training techniques. AI has proven helpful in analyzing medical images, and developing appropriate algorithms is necessary. Moreover, the scarcity of high-quality training data poses a significant challenge to developing automated medical image analysis. Although one motivation for automatic image processing is to relieve the burden of medical imaging data on human experts, more public access points to thoroughly defined medical imaging data are necessary. Contemporary DL algorithms can derive training insights from massive datasets, making them well-suited for augmenting the human visual system in medical image analysis. However, there are limitations to these models, such as binary classification, limited performance, and insufficient interpretability. Addressing these limitations can help develop more effective and reliable models for lung disorder detection.

The healthcare industry has recently made significant strides in leveraging digital technologies, particularly AI techniques such as ML and DL, to tackle various challenges [19–26]. DL, a subclass of AI that focuses on image processing of X-ray images and CT scans, has dramatically changed COVID-19 detection by processing multi-layer images in a single intersection. While many studies have been reported about automatically detecting COVID-19 by analyzing X-ray images with DL, the challenge lies in achieving overall detection accuracy to differentiate between normal illnesses, pneumonia, and COVID-19 more precisely. In recent years, convolutional neural network (CNN), an existing DL approach, has significantly improved medical image classification. Image processing could be utilized to create pre-trained neural network models for an automatic COVID-19 diagnosis system employing chest radiography imaging data as input images [5].

In most circumstances, standard CNNs detect the region of the lung’s primary nodule without considering the nodules’ neighboring tissues. The classification of lung illnesses is a complex task that requires extensive information to be extracted from medical imaging. Although CNN achieves adequate accuracy, the model’s performance may deteriorate when image features such as rotation, tiling, and other uncommon image orientations are present. Furthermore, the quality and quantity of training data [27, 28] are crucial for DL models [29–42], which can be influenced by overfitting and class imbalance. Utilizing a hybrid model that incorporates the attributes of different DL models used for item detection and classification is necessary. Ensemble DL models can improve the accuracy and resilience of lung disease categorization by integrating the benefits of multiple models and limiting the defects of individual models. Ensemble DL models can improve accuracy and reliability compared to a single model. Still, the effectiveness of the models often depends on the dataset; hence, there is still a need for research in developing new DL models for lung disease classifications.

The main contributions of this research paper can be summarized as follows:

1. A new hybrid framework named RVCNet is proposed by integrating the ideas of ResNet101V2, VGG19, and basic CNN models to detect COVID-19 using chest X-ray images. RVCNet employs stacking ensemble and model concatenation approaches to improve classification accuracy compared to existing hybrid DL models in lung disease detection.
2. Hyperparameter optimization is implemented in the feature extraction phase to ensure the model selects the most important features from the chest X-rays. This, combined with the addition of batch normalization, dropout, and dense layers in the classification phase, optimizes the model’s performance in terms of accuracy, specificity, recall, F1-score, and loss. Finally, Grad-CAM (Gradient-weighted Class Activation Mapping) is applied to visualize crucial regions in the X-ray images for classification by the proposed RVCNet.

The paper has the following sections: Section 2 includes a literature review; Section 3 contains materials and procedures; Section 4 describes an architecture overview; and Section 5 provides experimental results and discussion. Section 6 shows the explainability of the proposed model; Section 7 includes discussion; and Section 8 contains the conclusion.

## 2. Related works

Several research studies were conducted in view of the current success of DL networks in medical image classification, including COVID-19 [6–10], pneumonia [11–13], thoracic diseases [14], lung cancer [15, 16, 19], pulmonary edema [17], etc. Although several DL models show promising accuracy in classifying diseases, they are only partially implementable for some datasets. As a result, developing lung disease detection methods based on DL remains an interesting research issue. In the following, the existing studies are described in two sections: stand-alone and hybrid models.

### 2.1. Stand-alone models

Albahli et al. [22] simulated 15498 numbers of another three classes of X-ray images using pre-trained DenseNet, Inception-V3 and Inception-ResNet-V4. The DenseNet model showed a maximum accuracy of 92%, while the accuracies of Inception-V3 and Inception-ResNet-V3 were 83.47% and 85.57%, respectively. Apostolopoulos et. al., [23] used 1427 X-ray images, of which 224 images of COVID-19, 700 images of bacterial pneumonia, and 504 images of normal conditions present. This research used five different models, such as VGG-19, Inception, MobileNet-V2, Xception and Inception-ResNet-V2, to distinguish three classes. The accuracy varied from 92.85% to 93.48%, and the maximum accuracy was found using the VGG-19 model, which requires more improvement. Four categories of different lung disorders with abnormalities for identification include COVID-19, Pneumothorax, Tuberculosis, and Pneumonia, and along with healthy patients’ datasets, all include a total of 3500 CXR-images with varying sizes of input have experimented on eight pre-trained neural networks achieving an average accuracy of 97.2% by S. H. Karaddi et al., [25]. However, these diagnostic DL techniques did not require feature selection and extraction. Similarly, Hong, Min, et al. [26] also proposed a multiclass classification method by learning four classes from three types of lung diseases except for COVID-19. In this study, two types of datasets (US National Institutes of Health (NIH) with 10,000 PNG images [27] and Soonchunhyang University Hospital (SCH) with around 51 thousand TIF images) of healthy, pneumothorax, tuberculosis and pneumonia lung abnormalities were applied to six pre-trained models. Their accuracy was also compared with each other. Moreover, Chiranjibi Sitaula *et al*. [44] developed a new approach, Bag of Deep Visual Words (BoDVW), for classifying chest X-ray images in diagnosing COVID-19. The method effectively differentiated COVID-19 infections from other pneumonia-related infections, showcasing its potential in medical image classification.

### 2.2. Hybrid models

A new hybrid DL framework was proposed by S. Bharati et al. [18] named VDSNet, in which the NIH chest X-ray image dataset was used to detect lung disease with acceptable validation accuracy. The proposed VDSNet was composed of a pre-trained VGG data spatial transformation network (STN) with some CNN layers on a total 5606 number of samples. Another hybrid model was proposed by combining a capsule network with VGG-16 for the classification of lung carcinoma. Tandon et al., [19] named the model VCNet which reached a higher level of testing accuracy at around 99%. The model was proposed only for lung cancer diagnosis. Quan et al., [20] developed another hybrid model like previous frameworks known as DenseCapsNet, in which a DL framework was designed using CNN and a capsular network. Using 750 chest X-ray images, the accuracy of detecting COVID-19 was found to be 90.7%, which needed to be improved, as did the number of data samples considered in this work. Sharma et al., [21] developed a model using hybrid Inception-ResNet-v2 to distinguish three classes of CXR images, such as COVID-19-positive patients, pneumonia-affected, and normal patients, with an accuracy of 98.66%. The accuracy of the proposed technique was also compared to other DL, ML and transfer learning methods, but it could not be implemented in a commercial setting. Das et al., [24] created a model that demonstrated automated identification of COVID-19 by ensemble learning using a convolutional network utilizing 538 COVID-19 and 468 non-COVID-19 X-ray images. The accuracy was 91.6%, and DenseNet201, ResNet-50-V2, and Inception-V3 were also used to compare the accuracy with the suggested models. Tang et al. [37] proposed EDL-COVID, a DL framework using DCNNs for COVID-19 detection from chest X-rays, achieving 97.5% accuracy. The study had limitations, including a small dataset and a lack of patient diversity. Further research is needed for validation on larger, diverse datasets. C. Sitaula *et al*., [43] proposed DL-based methods for sentiment analysis on Nepali COVID-19-related tweets, focusing on the Nepali language. The authors introduced novel feature extraction methods and convolutional neural networks (CNNs) models that demonstrated robust and stable performance in sentiment classification.

### 2.3. Summary of stand-alone and hybrid models

These above studies [18–25, 37, 43, 44] are summarized in Tables 1 and 2. Related works are presented in Table 1 for stand-alone models and Table 2 for hybrid models.

**Table 1:** Summarization of related works for stand-alone DL models.

Ref	Method	Dataset & Classes	Performance Metrics	Limitations
[22]	Simulation on pre-trained DenseNet, Inception-V3, and Inception-ResNet-V4	Pneumonia: 5463, COVID-19: 490, Normal: 7966	Accuracy: DenseNet (92%), Inception-V3 (83.47%), Inception-ResNet-V3 (85.57%)	Done for three classes
[23]	VGG-19, Inception, MobileNet-V2, Xception, Inception-ResNet-V2	1427 X-ray images (224 COVID-19, 700 bacterial pneumonia, 504 normal)	Accuracy: VGG-19 (93.48%), others vary from 92.85% to 93.48%	Improvement required for VGG-19
[25]	Experiment on 8 pretrained neural networks	3500 CXR-images for identification of COVID-19, Pneumothorax, Tuberculosis, Pneumonia, and healthy patients (700 CXR-images in each class)	Average accuracy: 97.2%	No feature selection and extraction
[26]	CNN model-based multiclass classification technique	51,866 images (16,517 Normal, 15,840 Pneumonia, 8,230 Pneumothorax, and 11,279 Tuberculosis from Soonchunhyang University Hospital)	Highest classification accuracy of 96% when applied to Soonchunhyang University Hospital	When applied to NIH dataset then accuracy is only 85%
[44]	BoDVW (Bag of Deep Visual Words)	4 COVID-19 CXR image datasets (D1, D2, D3, and D4)	Classification accuracy: 82.00% (D1), 87.86% (D2), 87.92% (D3), 83.22% (D4)	Differentiating COVID-19 from other Pneumonia-related infections

**Table 2:** Summarization of related works for hybrid DL models.

Ref	Method	Dataset & Classes	Performance Metrics	Limitations
[18]	VDSNet (hybrid DL framework)	NIH chest X-ray image dataset for detection of lung disease (5606 images)	Validation accuracy: 73%	Uses sample dataset which has slightly lower validation accuracy, but much lower training time
[19]	VCNet (hybrid model combining capsule network and VGG-16)	Lung Image Database Consortium (LIDC), 2 Class (Cancer and Normal)	Testing accuracy: 99%	Proposed only for lung cancer diagnosis
[20]	DenseCapsNet (hybrid model with CNN and capsular network)	750 chest X-ray images for detection of COVID-19	Accuracy: 90.7%	Not enough data samples and accuracy not excellent
			F1 score: 90.9%	
[21]	Hybrid Inception-ResNet-v2	CXR images (Pneumonia, COVID-19, and Normal)	Accuracy: 98.66%	Class imbalance in the dataset which was tackled using SMOTE and weighted class balancing
[24]	Ensemble learning using a convolutional network	538 COVID-19 and 468 non-COVID-19 X-ray images	Accuracy: 91.6%	Insufficient accuracy and dataset size
[37]	EDL-COVID (Ensemble DL)	Chest X-ray images (COVID-19 positive and negative)	Overall Accuracy: 95%	Models might suffer from overfitting, high variance, and generalization errors caused by noise.
[43]	Ensemble CNNs Nepali COVID-19 tweets (NepCOV19Tweets)	COVID-19 3 classes (positive, neutral and negative)	Stable classification performance (domain-specific method: 61.5%, domain-agnostic method: 59.5%, pretrained fastText model: 68.1%)	Ignores sequential approach of tokens; potential improvement with LSTM, Word2vec, and GloVe embeddings

From the above discussion, it is evident that there are research gaps in the literature that must be filled in order to improve DL-based lung disease diagnosis even further. The majority of DL models are constructed and trained on specialized datasets that may not sufficiently reflect varied populations in terms of demographic characteristics. To minimize biased predictions and increase the overall performance of lung disease detection systems, DL models must be robust and generalizable across different populations. To improve the generalization and accuracy of lung disease detection models, research efforts are necessary to build robust DL architectures or preprocessing strategies that can handle noisy and low-quality medical images. Hence, more research is required to identify and categorize lung diseases in the case of new and large datasets. To propose a DL-based lung abnormality detection model, the adequate number of training and testing data capability of distinguishing between normal, COVID-19 positive cases, non-COVID lung infection and viral pneumonia must be present with the highest possible accuracy. In conclusion, by addressing the challenges encountered in previous works and advancing the capabilities of existing models, a new classification and prediction deep neural network must be developed, which aims to enhance the accuracy and effectiveness of lung disease prediction from chest X-ray images. Hence, this paper proposes a new deep neural network framework for predicting lung diseases from the X-ray images of the chest.

## 3. Materials and methods

### 3.1. Dataset availability

**Fig 1:**
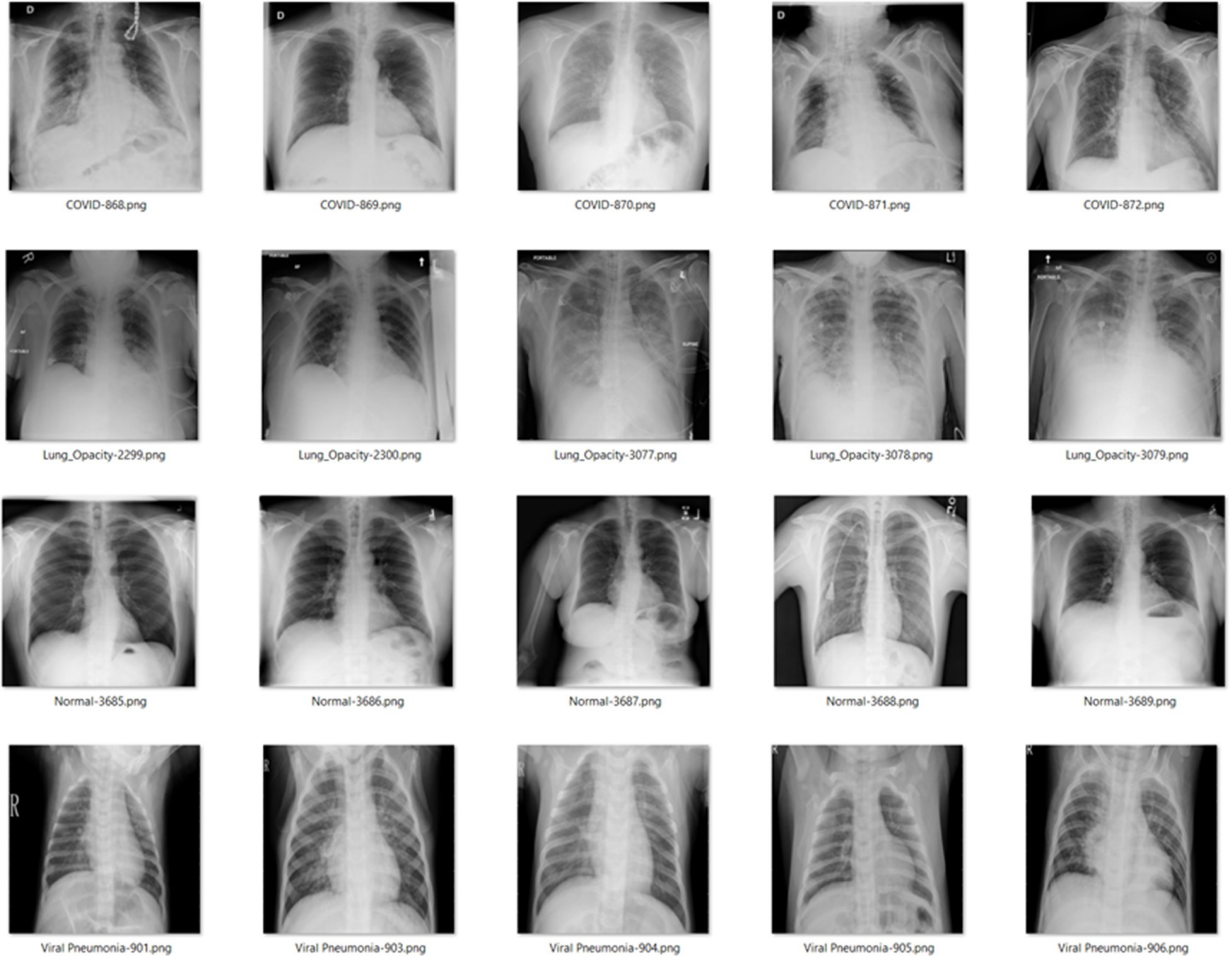
Radiography X-ray image from the dataset.

Nowadays, a huge open-source dataset of chest radiography is available in the Kaggle repository. In this research, X-ray scanned images belong to four categories. The hybrid framework is capable of detecting healthy patients, COVID-19-positive cases, lung opacity (Non-COVID lung infection) and viral pneumonia-affected patients in the four categories with better accuracy. These four types of radiography images contain a total of 2514 X-ray samples, out of which pneumonia-affected and normal patient X-rays were 243 (9.67%), and 1115 (44.35%) samples each, 442 (17.58%) samples COVID-19 affected and finally 714 (28.4%) samples for Lung Opacity were publicly available datasets which got the winner dataset award by Kaggle Community [28]. Fig 1 shows some of the radiography X-ray samples from the dataset. The experiments were done for holdout method as well as cross-validation method. As part of holdout method, the whole dataset was split into 90% for training and 10% for testing samples as shown in Table 3. This division was done to keep consistency with some literature for ML to achieve a good balance between learning capacity and validation of the model’s performance on new data. This data split helped the model to effectively learn patterns and features while still retaining a portion for testing its ability to generalize to unseen data. Furthermore, the full dataset was tested with 10-fold cross-validation, which separates the data into ten subgroups. After being trained on the previous nine subsets for each subset, the model was evaluated on the most recent subset. The procedure was repeated ten times, with one test set performed for each subgroup. The model ensured robust learning and reliable validation by adopting established ML practices, including data splitting and 10-fold cross-validation. Due to technical limitations and constraints associated with handling large volumes of data within the framework employed in this research, performing 10-fold cross-validation in a single run was not feasible. Consequently, each iteration of the cross-validation was executed separately on the whole dataset.

**Table 3:** Summary of our experimental dataset splitting into training and testing images.

Class Labels	Class Name	Training image	Testing image
0	COVID-19	408	34
1	Lung Opacity	640	74
2	Normal	1000	115
3	Viral Pneumonia	214	29
Total images		2262	252

### 3.2. Dataset preprocessing and augmentation

Data preprocessing in ML is the process of transforming the data of a dataset into an efficient format. Data augmentation is a strategy for increasing the data diversity of the training model while reducing the number of training samples. However, data augmentation allows the model to learn a wider range of features, which not only increases the size of the dataset but also helps prevent model overfitting. The normalization of each pixel value was accomplished by shifting from the spectrum [0, 255] to a standardized [0, 1] range, which was helped by a scaling factor of 1./255. The Keras ImageDataGenerator class was used for image resizing and enhancement, converting the data from 256x256 dimensions to the CNN-required input size. Keras’ ImageDataGenerator tool’s main advantage was its real-time data augmentation functionality. Each epoch while the model was training, this program generated slightly altered, or “augmented,” versions of the original images. The augmentation techniques and parameters (e.g., rotation, translation, scaling, and flipping) determined the diversity and variability in enhanced samples. For this study, we used a 15-degree rotation range, a 0.2 latitude and height shift range, a 0.2 zoom range, horizontal and vertical flips, and a fill new pixel mode as the “next” option to get better results. While the number of epochs (EPOCHS) and batch size (BATCHSIZE) determine how frequently and how many of these augmented samples were presented to the model during training, the size of the original dataset remains constant. These parameters, instead, influenced the frequency and batched grouping of the augmented samples processed. For a more in-depth look, our model ran 25 epochs, processing 64 images in each batch and 35 batches in each epoch.

### 3.3. Stacking ensemble

Ensemble learning is a method that combines multiple models to enhance machine learning outcomes. Different ensemble approaches such as stacking, boosting, bagging, and voting all perform differently depending on the dataset, the complexity of the task, the quality of the individual models, and other factors. There is no one-size-fits-all solution. Each ensemble strategy has its own set of benefits and drawbacks. Stacking has several advantages, including the ability to mix forecasts from several base models. Stacking can capture higher-order interactions between models by training a meta-model on their predictions. Stacking responds to the strengths and weaknesses of each underlying model by assigning different weights to its predictions. This technique adeptly uncovers intricate patterns and mitigates both bias and variance, frequently yielding enhanced results compared to singular models. Alternative ensemble methodologies, like voting, bagging, and boosting, may not sufficiently tackle the complexity of the problem or could be affected by the selection of base models and their hyperparameters. Hence, this paper considers stacking algorithm to form RVCNet.

Stacking is an ensemble technique that assembles a variety of well-performing algorithms to merge their best predictions on the same dataset. The stacking ensemble architecture consists of two or more base models, with level-0 base models fitting the training data and compiling predictions. Level-1, known as the meta-model, is trained on the predictions made by the base models, taking their output as input. This framework enables the creation of a stacking ensemble of multiple machine-learning models.

### 3.4. Performance metrics

In this paper, the confusion matrices, training and testing accuracy, loss, sensitivity or recall, specificity, precession, F1 score, and ROC and AUC curves were used to evaluate the proposed models. A confusion matrix is a tabular representation of a model’s performance predictions, with each item representing the number of predictions made by the model that classified a class correctly or incorrectly. Accuracy is the calculation of all the truly recognized cases as (TP + TN) / (TP + TN + FP + FN) where TP is the number of true positives, TN is the number of true negatives, FP is the number of false positives, and FN is the number of false negatives. It is determined because the range of all true predictions is divided by the whole number of the dataset. Sensitivity or recall is used to assess the completeness of a classifier by detecting True Positives denoted as TP / (TP + FN). It is calculated by dividing the total number of positives by the number of real positive results. The number of true negative values divided by the total number of true negative and false positive data is used to calculate specificity expressed as TN / (TN + FP). Precision is defined as having a positive predictive value (TP / (TP + FP)). Precision is calculated by dividing the total number of positive forecasts by the number of real positive identifications. Precision and recall combinations F1-score as 2*(Precision*Recall)/(Precision+Recall). The ROC curve is a receiver work function graph that indicates the overall performance type of a version based mostly on factors such as true positive and false positive rates. The area under the curve (AUC) is calculated for both training and testing epochs.

## 4. Proposed architecture

The following subsections will briefly describe the functionalities and mathematical operations of the proposed RVCNet model. Note that RVCNet combines the fine-grained details collected by VGG, the deep architecture of ResNet, and the broad feature extraction capability of CNN to achieve superior performance. Given that RVCNet combines the ideas of CNN, VGG, and ResNet models, a short discussion of custom CNN, VGG, and ResNet is given first, and then RVCNet is described.

### 4.1. Custom CNN architecture

CNN consists of three main layers: convolutional, pooling (average, max, etc.) and fully connected. There are many popular architectures for CNN feature extraction and classification, such as LeNet, VGG, ResNet, Xception, Inception, MobileNet etc., which can handle huge amounts of datasets. The purpose of convolution layers in the neural network is to extract specific features from the input images. The convolutional layers have different types of weighted filters to produce different feature maps by a convolution operation. Convolution neural networks also perform some operations in pooling layers; from them, average pooling entails calculating an average for every patch on the feature map. The initial phases in the CNN convolution and pooling divide the image into features and evaluate each one independently. The output of this process is sent into a fully connected neural network structure, “flattens” it into a single vector that may be used as an input for the next step. Then it passes through various layers before ending at the fully connected output layer, which decides the final classification with each label. During each training phase, a generalized technique increases accuracy by preventing a model from being overfitted. Dropping off neurons can be applied to hidden neurons but does not consist of forward and backpropagation in neural networks. This process temporarily does not allow some neurons to emit with a certain probability, and other than that neuron’s probability of, [32] both learning and training have been done. The dropout layer should be placed before the ReLU or after the other activation function.

A neuron’s activation status is decided by an activation function. This indicates that during the prediction phase, it will employ more straightforward mathematical operations to determine if the neuron’s input to the network is crucial or not. The rectified linear activation function (ReLU) is a piecewise linear function that outputs zero if the input is negative and the input directly if the input is positive. A type of activation function known as a Leaky Rectified Linear Unit (Leaky ReLU) is based on a ReLU but has a small slope for negative values rather than a flat slope. Softmax is implemented immediately before the output layer using a neural network layer. The number of nodes in the Softmax layer must be the same as that in the output layer. A custom CNN model is used in this research; the architecture contains three convolutional layers followed by a max pooling layer.

### 4.2. VGG

The Visual Geometry Group (VGG) based neural network is widely regarded as one of the most popular pre-trained models for image classification in the field of DL [29]. The VGG network is characterized by a high degree of uniformity and simplicity in convolution and max pooling operations. One of the unique design principles of the VGG network is the doubling of the number of filters used on every stack of convolution layers. VGG-16 and VGG-19 variants share the same design principles, with the latter having a few additional convolution layers. In each stage of the VGG network, 3×3 small filters are employed to decrease the number of parameters required for image classification. The network also uses ReLU activation functions in all hidden layers, which replace all negative values with zero. In the development of RVCNet, the VGG19 variant is considered.

### 4.3. ResNet

ResNet101V2 [30] is a form of a neural network having residual networks with a 101-layer CNN. The concept of ResNet came from having alternative ways that bypass at least one layer. This design is inspired by VGG19’s stacked network, after which a shortcut connection is applied as a residual connection, resulting in bypass links all over the architecture. The advantage of the architecture is to tackle the vanishing gradient problem and train thousands of layers. Table 4 shows the architecture of ResNet version 101, the details can be found in [34].

**Table 4:** Deep residual learning for image recognition [34].

Layer Name	Output size	Layer 101
Conv1	112X112	Kernel 7X7, filter 64, stride 2
Conv2_X	56X56	Kernel 3X3, max pool, stride 2
		$\left[\begin{array}{cc} 1x1 & 64\\ 3x3 & 64\\ 1x1 & 256 \end{array}\right]X3$
Conv3_X	28X28	$\left[\begin{array}{cc} 1x1 & 128\\ 3x3 & 128\\ 1x1 & 512 \end{array}\right]X4$
Conv4_X	14X14	$\left[\begin{array}{cc} 1x1 & 256\\ 3x3 & 256\\ 1x1 & 1024 \end{array}\right]X23$
Conv5_X	7X7	$\left[\begin{array}{cc} 1x1 & 512\\ 3x3 & 512\\ 1x1 & 2048 \end{array}\right]X3$
	1X1	Average pool, 1000-d fc, softmax

### 4.4. Proposed RVCNet

The proposed RVCNet algorithm is illustrated in this subsection. For that, concepts of ResNet and VGG are used in part of the architecture with another basic custom CNN model for feature extraction, utilizing stacking ensemble and concatenation for improved accuracy. As part of RVCNet, VGG is used for capturing fine-grained visual information; however, it may struggle with highly deep structures. ResNet uses skip connections to allow the network to acquire residual characteristics, which aids in the training of very deep network topologies. ResNet, on the other hand, may be less effective at capturing fine-grained features. CNNs have shown good performance in a range of applications, including lung disease classification. Combining these three models maximizes their strengths while limiting their weaknesses. Next, the output was sent to classification layers containing flattening and batch normalization along with dense and dropout layers with a number of filters and activation functions.

**Fig 2:**
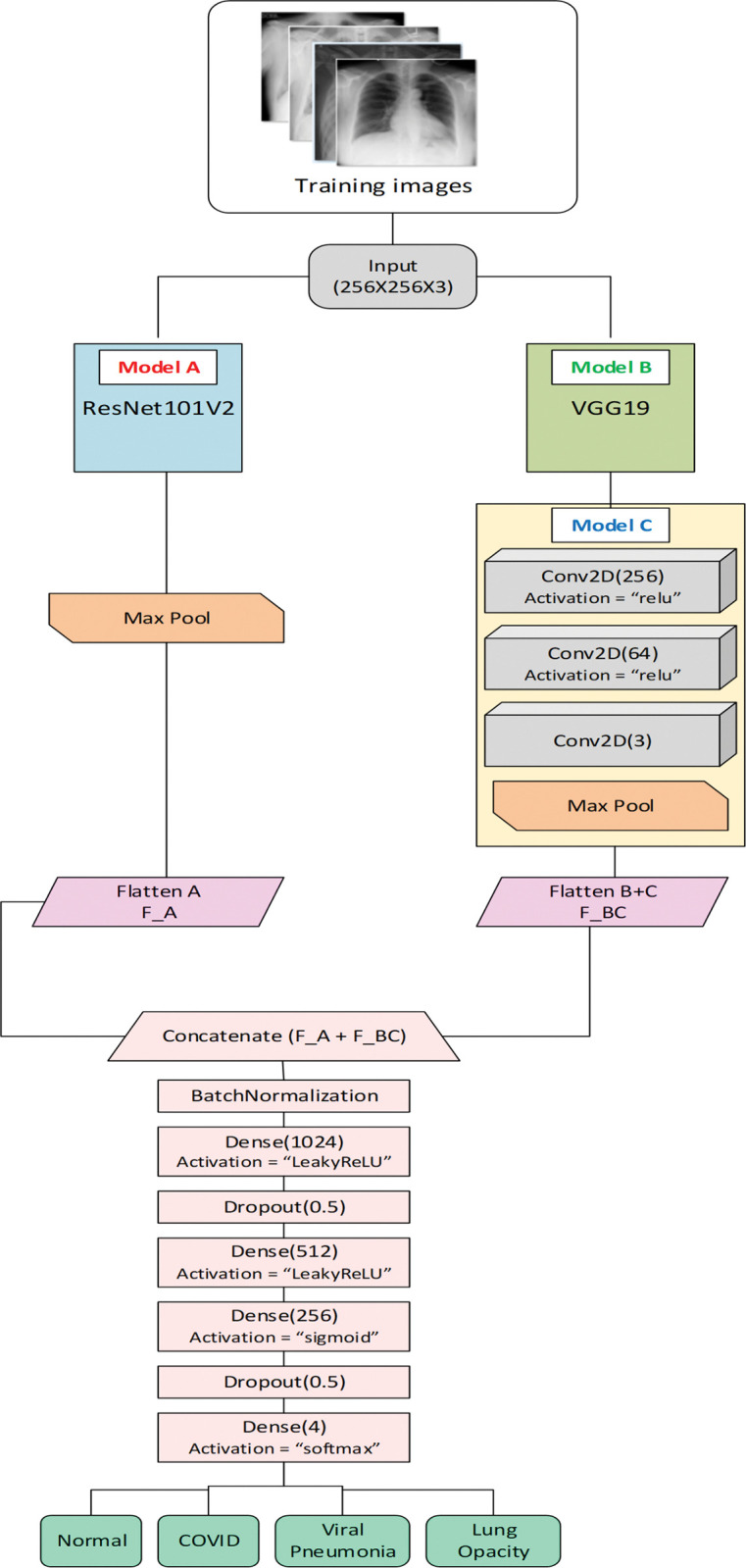
Proposed RVCNet architecture.

Fig 2 presents the architecture of the proposed RVCNet for image classification. The input training images, with an RGB channel for each image, have an initial size of 256×256×3. In the first block of the architecture, the same input is fed into two pre-trained models, namely ResNet101V2 (Model A) and VGG19 (Model B). The pretrained ResNet101V2 model up to the Activation layer is used in the upper left portion of the architecture, and an additional max pooling layer is added with filter size 2×2 and stride 2 with non-trainable parameters. The final output size becomes 4×4×2048, which is then passed to a flatten layer, denoted as F_A. In parallel to Model A, VGG19 is applied to the training image, and its output up to its last max pooling layer is produced as 8×8×512. This output is used as input to another Model C, which contains three convolutional layers followed by a max pooling layer. The stacking ensemble ML algorithm is used to stack Model C (VGG19) over the output of Model B (customized CNN). For the trial, other stacking combinations were actually considered. However, the combinations of Model B over the output of Model C, and the stacking of model C over the output of model A (ResNet) showed less accuracy and more loss compared to the proposed architecture of RVCNet. The first convolutional layer contains 256 filters with kernel size (5,5) and stride (1,1) with the same padding. The output is then fed into a second convolution layer with 64 filters, each having a kernel size of (3,3), and then the final convolutional layer has a number of filters of 3 with the same parameters as the initial convolutional layer. The three layers are composed of a “ReLU” activation function with a trainable parameter. Finally, the output is used as input to a max pooling layer with kernel size (2,2) and then sent to another flatten layer named F_BC. The extracted features of F_A and F_BC are merged using an additional Keras layer called Concatenate() layer, which simply adds the outputs of the flatten layers. The concatenated features are added to a BatchNormalization layer in the classification stage. The output is then sent to a dense layer having 1024 filters with a “LeakyReLU” activation followed by a dropout layer keeping 0.5. Another two dense layers are added sequentially with 512 and 256 filters, one with “LeakyReLU” activation and another with a “sigmoid” activation function. A final dropout layer is used as before, and then a “softmax” layer is used in a dense layer for classifying into four classes COVID, Normal, Viral Pneumonia, and non-COVID lung infection samples.

**Fig 3:**
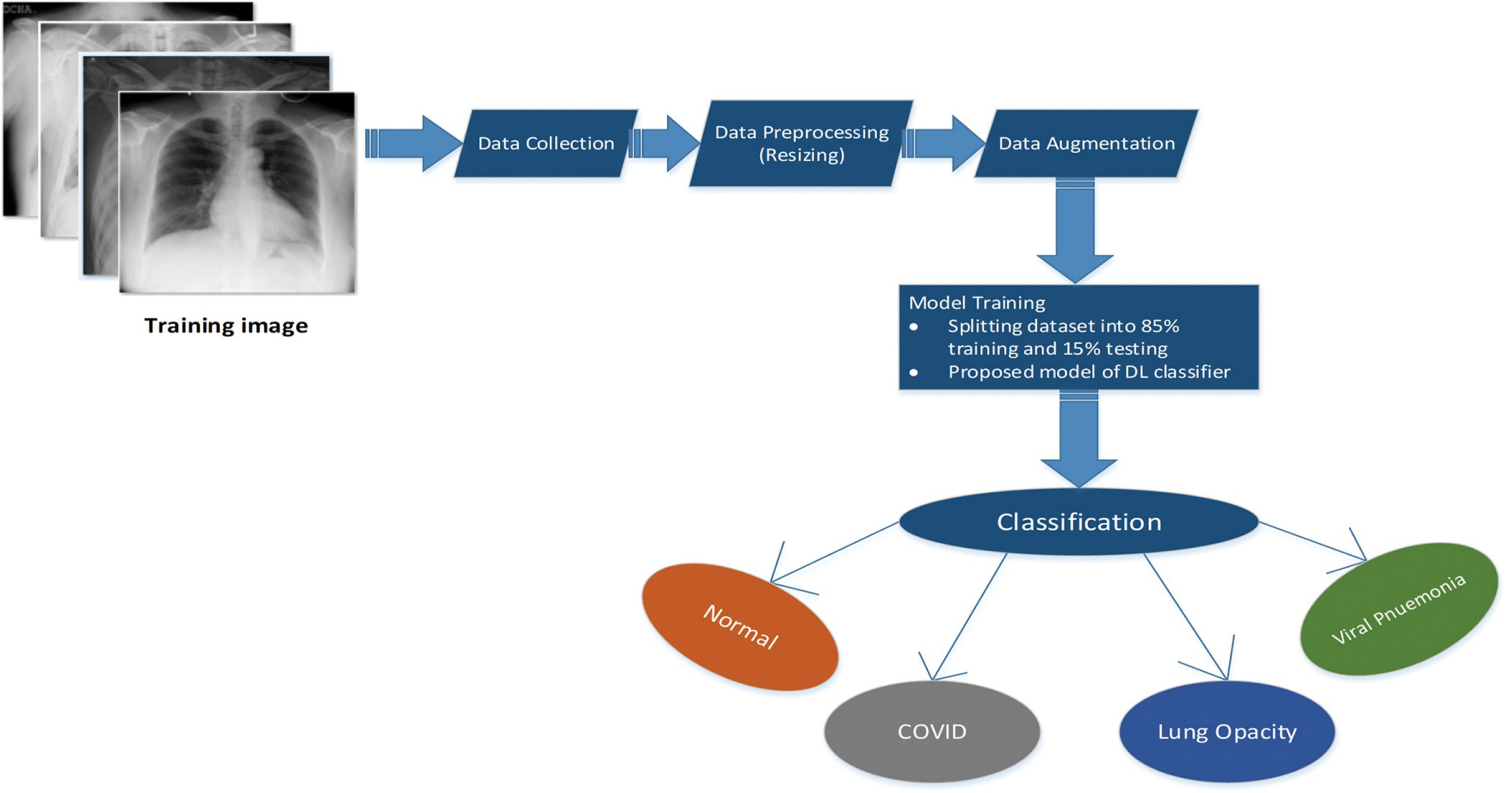
Basic methodology of the overall system.

Fig 3 shows the overall process of the proposed method with some stages of the experiments. In the first stage of the process, images are collected from the Kaggle dataset [28] with different shapes. In the data preprocessing stage, the images are labelled according to the four categories and resized into 256×256. Data augmentation is then applied with some parameters. The fourth stage consists of the implementation of CNN models. A hybrid model, RVCNet, is implemented for training, and performance is analyzed in this stage for classifying normal, COVID-19 positive cases, non-COVID lung infection, and viral pneumonia classes in this study. The model was trained separately for choosing the best values for three batch sizes (16, 32 and 64), learning rates (0.0008, 0.002, 0.007 and 0.05) and optimization functions (RMSprop, SGD, Nadam and Adam) in Table 5. The data is trained up to 25 epochs, and the best accuracy of the RVCNet model is shown within 25 epochs, with a batch size of 64 and an initial learning rate of 0.002. The overall performance is analyzed in terms of accuracy, recall, precision, AUC, and F1 score.

**Table 5:** Performance comparison with different lea rning rates, batch sizes and optimizer functions.

Metrics	Performance	Accuracy (%)	Loss (%)	Recall (Sensitivity) (%)	Precision (%)
Learning Rate (for Batch Size: 64)	0.0008	82.94	49.49	95.64	80.16
	0.007	73.81	66.25	92.38	69.05
	0.05	87.75	41.84	97.03	86.67
Batch Size (for Learning Rate: 0.002)	16	87.70	36.07	97.76	87.70
	32	89.29	35.14	86.45	88.11
Optimizer Model	RMSprop	86.11	45.00	86.11	86.80
	SGD	90.08	27.73	98.55	89.29
	Adam	84.94	50.07	95.64	82.56
**Metrics for RVCNet**	**LR: 0.002**	**91.27**	**27.90**	**98.30**	**90.39**
	**BS: 64**				
	**Opt: Nadam**				

## 5. Experimental results

This section describes the experimental results based on performance metrics. The implementations of the architectures were performed by Google Collaboratory, known as Google Colab, which is an open-source cloud-based platform to write and execute arbitrary Python code to be used for experimental purposes. The code runs on an NVIDIA Geforce RTX 3060 GPU platform having 12.0 GB RAM in processor Intel(R) Core (TM) i5-8265U CPU @ 1.60GHz 1.80 GHz. Furthermore, ML libraries such as Numpy, Scipy, Scikit-learn, matplotlib, and others were utilized in the experiment. Numerous optimizers based on DL and the CNN framework were investigated to increase the accuracy of the proposed model. In this experiment, an optimizer named Nadam, with a learning rate of 0.002, was utilized. Finally, the model was saved and compiled. In case of 10-fold cross-validation for validation of the purpose model, the results are observed for individually executed iterations in Table 6. After applying 10-fold cross-validation and randomly selecting 10% of the dataset for testing, it is observed that the results are quite similar. However, in the process of 10-fold cross-validation, the results are obtained through manual observation of each iteration, repeated 10 times due to hybrid architecture. When the same dataset was processed through RVCNet, the computational times were marginally longer than when the dataset was processed through stand-alone models of ResNet101V2, VGG19, and CNN over VGG19. In Table 7, multiple runs for the model are observed, and with these values, a summary table (Table 8) is made with mean, standard variation, best, worst, count, and total for the given metrics across all runs. Combining popular DL models is not commonly done, particularly when considering more substantial models like DenseNet, InceptionV3, and NASNet. These advanced models are characterized by their depth and task-specific training. Trying to stack them in order can lead to problems like overfitting, high computer costs, and hard training. Because of these issues, the ResNet and VGG methods were chosen for the lung disease dataset. However, the overall classification accuracy and other performance metrics for RVCNet were better than the stand-alone models. Table 9 depicts the comparison between all models mentioned in this paper and also simulated by other popular pre-trained models (InceptionV3, NASNet, DenseNet169, DenseNet201, etc.) to compare their performances with RVCNet on the same dataset.

**Table 6:** 10-fold cross-validation metrics of individually executed iterations.

Fold	Accuracy	Loss	Precision	Recall
1	0.9841	0.0826	0.9841	0.9994
2	0.9722	0.3145	0.9127	0.9984
3	0.9484	0.1626	0.9405	0.9962
4	0.9484	0.3509	0.8849	0.9960
5	0.9562	0.1777	0.9562	0.9936
6	0.9163	0.2585	0.8964	0.9874
7	0.7778	0.5798	0.7698	0.9433
8	0.8407	0.4238	0.8208	0.9779
9	0.8649	0.5778	0.8331	0.9615
10	0.8538	0.6041	0.8498	0.8692
Average	0.9063	0.3535	0.8848	0.9723

**Table 7:** Results of multiple runs for RVCNet.

Metric	Run 1	Run 2	Run 3	Run 4	Run 5
Model Accuracy	0.913	0.905	0.897	0.902	0.900
AUC	0.923	0.912	0.906	0.915	0.904
Loss	0.279	0.298	0.327	0.311	0.302
Precision	0.904	0.893	0.878	0.891	0.889
Recall	0.983	0.971	0.959	0.964	0.969
F-1 score	0.942	0.929	0.919	0.926	0.925

**Table 8:** Summary table with the mean, standard variation, best, worst, count, and total for the given metrics across all 5 runs.

Metric	Mean	Standard variation	Best	Worst	Count	Total
Model Accuracy	0.9034	0.0058	0.9130	0.8970	5	4.517
AUC	0.9120	0.0087	0.9230	0.9040	5	4.560
Loss	0.3034	0.0188	0.2790	0.3270	5	1.517
Precision	0.8910	0.0107	0.9040	0.8780	5	4.455
Recall	0.9692	0.0080	0.9830	0.9590	5	4.846
F-1 score	0.9282	0.0095	0.9420	0.9190	5	4.641

**Table 9:** Comparison of proposed architecture with separate models.

DL Models	ResNet101V2 (Input 256×256)	VGG19 (Input 331×331)	CNN over VGG19 (Input 331×331)	InceptionV3 (Input 256×256)	NASNetLarge (Input 331×331)	DenseNet 169 (Input 224×224)	DenseNet 201 (Input 224×224)	RVCNet
Model Accuracy	0.814	0.798	0.791	0.8571	0.8906	0.8730	0.8810	**0.913**
AUC	0.822	0.836	0.793	0.8739	0.8871	0.8710	0.8880	**0.923**
Loss	0.5433	0.491	0.644	0.4392	0.3479	0.3629	0.3698	**0.279**
Precision	0.786	0.750	0.614	0.8254	0.8594	0.8571	0.8492	**0.904**
Recall	0.949	0.958	0.922	0.9632	0.9671	0.9745	0.9750	**0.983**
F-1 score	0.862	0.841	0.787	0.8889	0.9101	0.9120	0.9078	**0.942**
Computational time for training a model. (In seconds)	1183.631	1141.807	1130.667	1156.2	1205.331	1140.23	1085	**1261.342**

**Fig 4:**
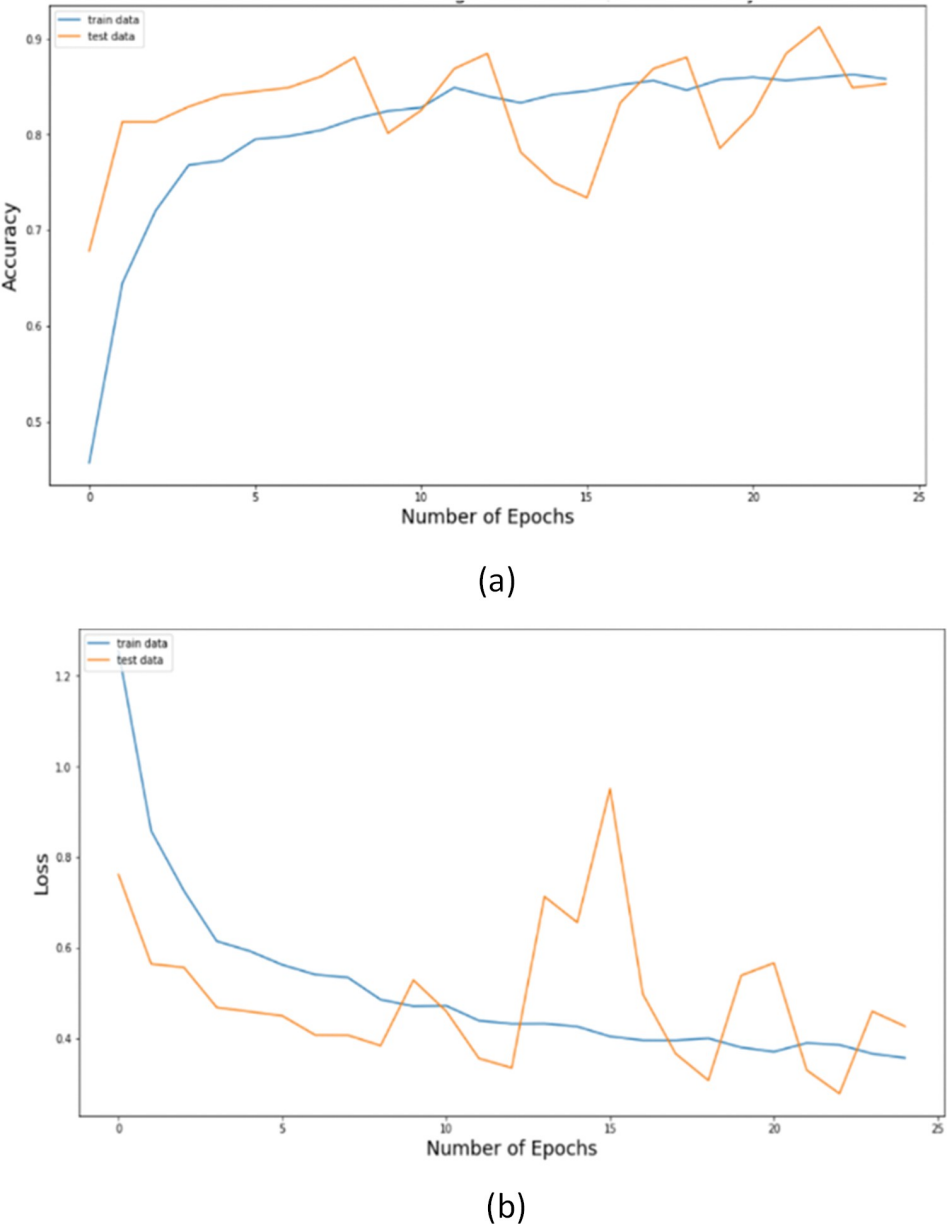
Training and Testing analysis of RVCNet within 25 epochs: (a) for accuracy (b) for loss.

**Fig 5:**
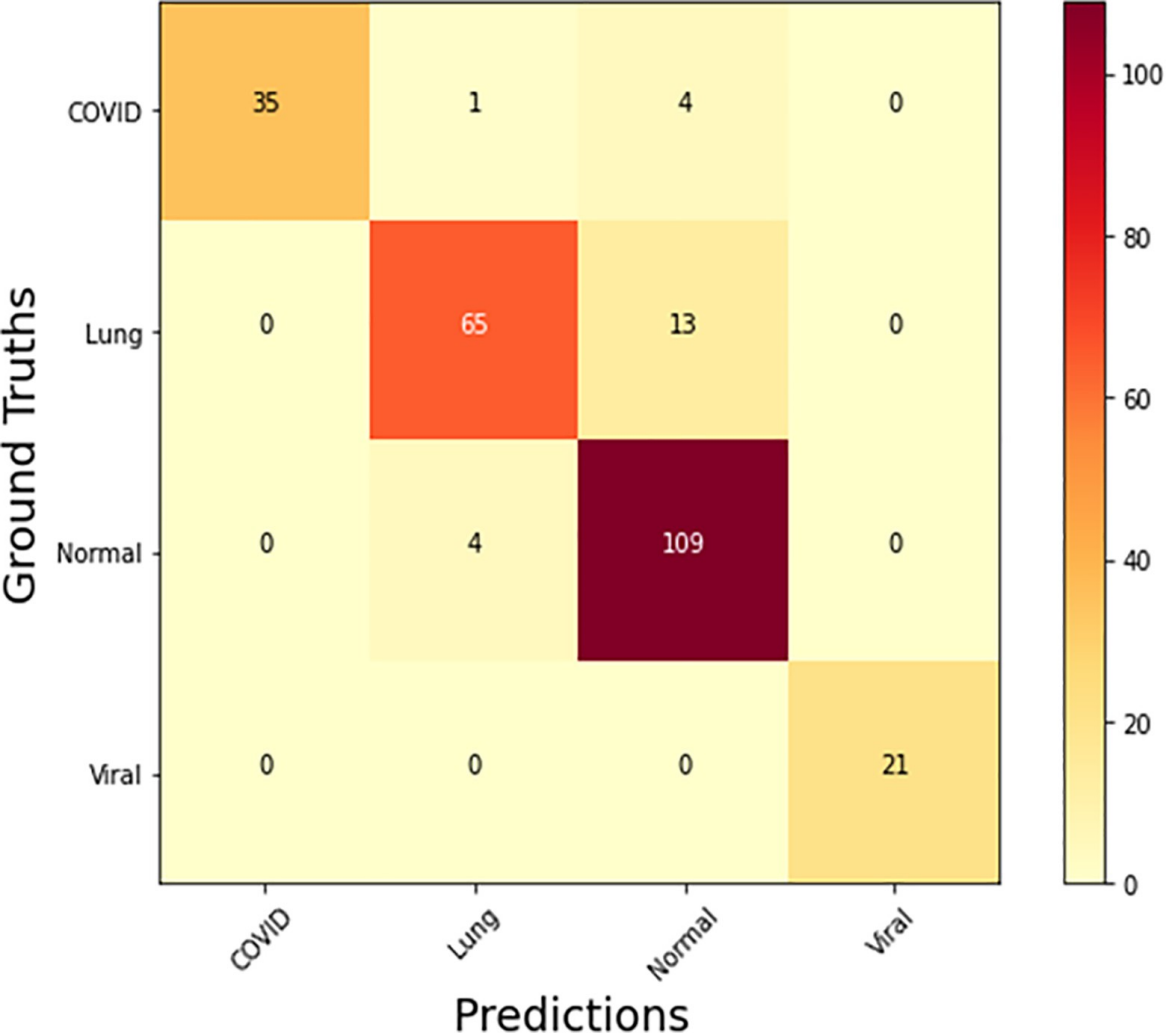
Confusion matrix of proposed RVCNet.

The proposed hybrid model is compared against competing models using different confusion matrix-based measures. The proposed model, as appears in Fig 4, achieves higher accuracy and lower loss values at the 25^th^ epoch. In order to avoid misclassification of diseases, it is recommended in medical research to minimize all false positive and false negative instances. Because they can be harmful to society, it is suggested that possibly the number of incorrectly found occurrences be minimized. The dataset is divided into four levels, and we calculated true positive (TP), false positive (FP), true negative (TN), and false negative (FN) to illustrate how many instances were either truly affected or incorrectly detected, as shown by the confusion matrix in Fig 5. In certain circumstances, people are not impacted by Lung Opacity, COVID-19 or viral pneumonia but are nonetheless affected. Furthermore, in certain situations, patients were affected by Lung Opacity, COVID-19, or viral pneumonia, although the findings showed that they were not.

**Fig 6:**
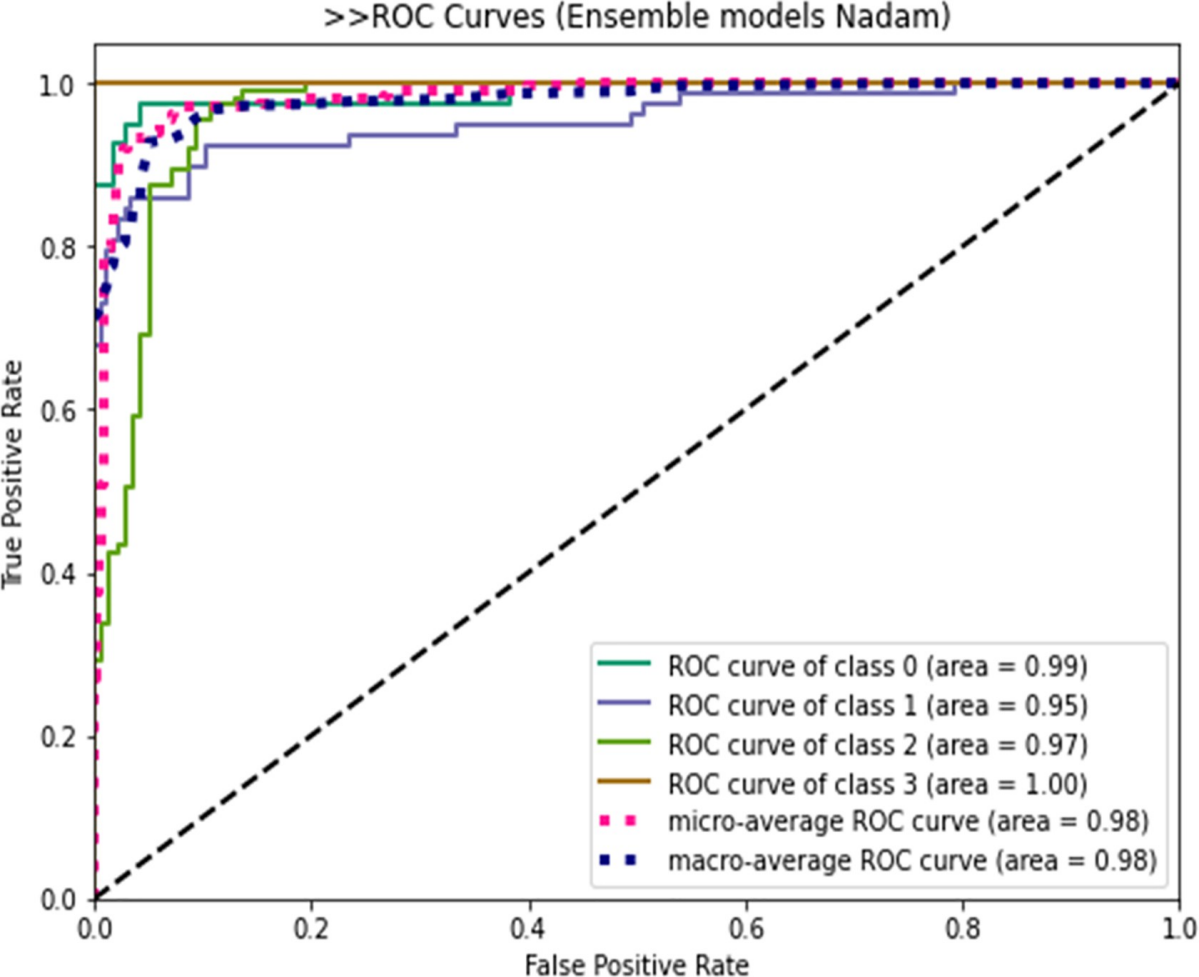
The ROC curves of the proposed RVCNet.

The ROC curve depicts the model’s classification performance in terms of true positive and false positive rates in two scenarios. Fig 6 shows the ROC curve for areas of the viral-pneumonia positive case (class 3) that performed considerably better on the hybrid model compared with the other three classes. The classification accuracy of the model is 0.9127, while its AUC, precision, recall, and overall loss are 0.9231, 0.9048, 0.9830, and 0.2785, respectively.

The RVCNet framework’s performance was then examined on a fully balanced dataset. A new dataset was built for this purpose by taking 200 samples from our primary dataset for each class. For this balanced number of image classes, RVCNet achieved an acceptable classification accuracy of 87.50%. This accuracy value was higher than that obtained for stand-alone VGG19, CNN, and ResNet101v2. This suggests that RVCNet is suitable even for a fully balanced dataset.

## 6. Model explainability

**Fig 7:**
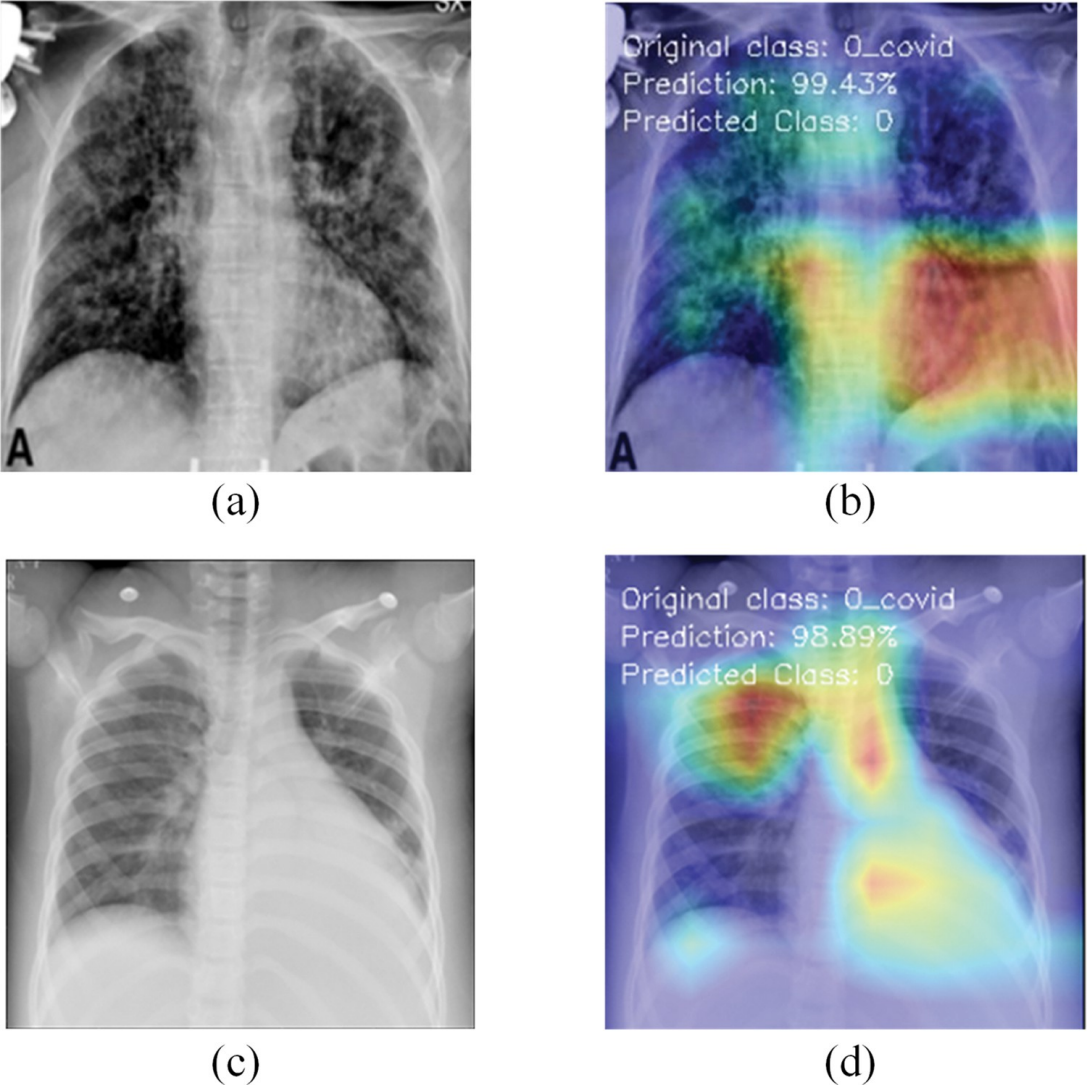
Visualization for COVID-19 X-ray test images with Grad-CAM heatmaps: (a, c) original images; (b, d) corresponding Grad-CAM heatmaps.

**Fig 8:**
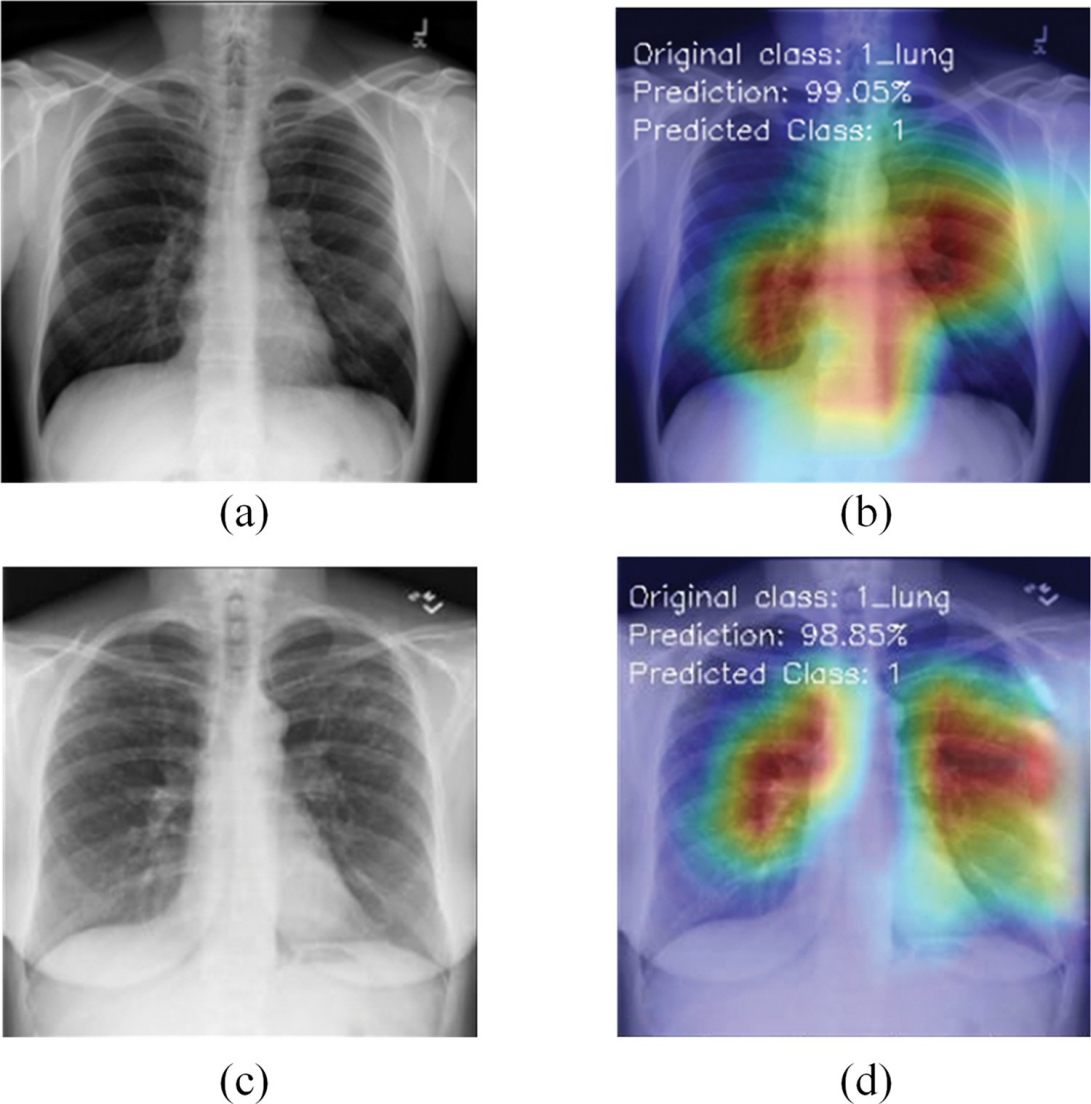
Visualization for Lung Opacity X-ray test images with Grad-CAM heatmaps: (a, c) original images of four samples; (b, d) corresponding Grad-CAM heatmaps.

**Fig 9:**
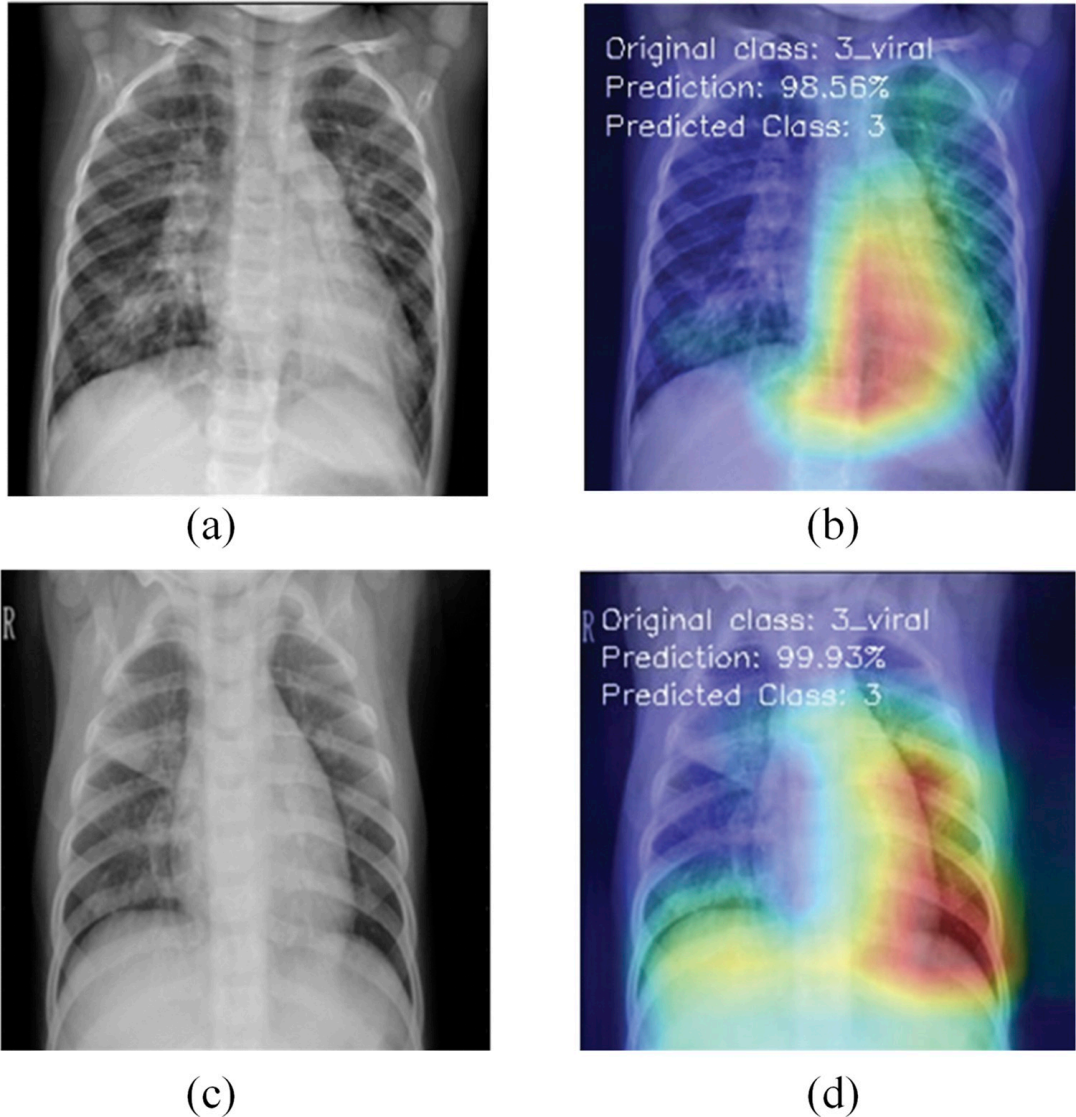
Visualization for Viral Pneumonia X-ray test images with Grad-CAM heatmaps: (a, c) original images; (b, d) corresponding Grad-CAM heatmaps.

This section discusses the explainability of the proposed RVCNet architecture. For this, Grad-CAM scheme is used to visualize the areas of the model that are activated during the decision-making process. The Grad-CAM heatmap is illustrated in bright red hues to underscore the vital regions of an image. This approach applies to the final convolutional layer and profoundly influences the classification decision of the model. Figs 7–9 present the original and preprocessed images of COVID-19, viral pneumonia, and lung opacity test cases. These figures also include the corresponding heatmaps generated for those images by the RVCNet model, for each class. Additionally, the accurately predicted class and prediction percentage are also provided on the heatmap. While most recent research primarily [45, 46] uses heatmaps to identify the areas of the lungs affected by COVID-19, this study (as shown in Figs 7–9) demonstrates that Grad-CAM aids efficient identification of damaged lung areas, which is paramount for accurate and prompt diagnosis and treatment. The visualization of RVCNet’s results, which shows how Grad-CAM correctly identifies the affected areas, gives doctors more confidence in the system and makes them sure that it can make accurate and fast evaluations.

## 7. Discussion

The proposed RVCNet is a hybrid DL-based model with improved accuracy compared to existing models applied on the same dataset and multiclass lung disease detection. In this section, RVCNet is compared with some existing models. A DL model’s performance is determined by the dataset applied, and the performance of several popular DL models can be fairly compared when applied to the same dataset. According to the literature, DL models are applied to various datasets; hence, the results of RVCNet in this paper cannot be directly compared to those models. As a result, we first compare RVCNet to several models for other datasets considered in previous studies. Then, for the dataset under consideration in this paper, we compare RVCNet to others. Table 10 presents the performance of RVCNet with some existing studies [47–50] that consider partially or fully the samples of two datasets [51, 52]. For fair comparison, the results for RVCNet are also provided for the samples of the same dataset [51, 52]. It can be seen from Table 10 that the RVCNet has a comparable performance to other models reported in [47–50]. Next RVCNet is compared to some other models for the same dataset. Table 11 compares the RVCNet model with the existing models for the same dataset for classification of COVID, viral pneumonia, lung opacity, and healthy persons. Results indicate that RVCNet outperforms existing models when applied to the same dataset.

**Table 10:** Comparison of RVCNet with others for several data samples.

Ref.	Dataset	Method	Performance Metrics
[47]	Normal: 1203, Bacterial Pneumonia: 660, Viral Pneumonia: 931, COVID-19: 290. [51, 52]	CoroNet	Accuracy: 89.6%, Recall: 89.92%, Precision: 90%, F-measure: 89.8%
[48]	COVID-19 and Others [51, 52]	nCOVnet	Accuracy: 88.10%
[49]	All samples from [51, 52]	VGG-16	Acc: 79.58%, 85.43%
		VGG-19	Acc: 74.84%, 83.83%
[50]	Total 1638 (Normal, Bacterial Pneumonia, Viral Pneumonia, COVID-19) [51, 52]	DL based Multi-scale bag of deep visual words (MBoDVW)	Accuracy: 88.88% | F-Score: 89.40% | Precision: 88.58% | Recall: 89.40%
Proposed Model	Total: 937, Normal: 413, Bacterial Pneumonia: 275, Viral Pneumonia: 108, COVID-19: 141. [51, 52]	RVCNet	Accuracy: 89.70% | AUC: 89.04% | Precision: 87.17% | Recall: 96.40%

**Table 11:** Comparison of RVCNet with others for classification of COVID, viral pneumonia, lung opacity, and healthy persons.

Ref	Dataset	Method	Performance Metrics
[22]	**Radiography X-ray dataset (COVID, Viral pneumonia, Lung opacity, healthy**)	Inception-ResNetV4	Accuracy 85.57%
[33]		MVGG	Accuracy 89.8%
[39]		Pre-training fine-tuning of DL model on chest radiographic images using DenseNet-121 by joint learning	Average AUC of 82.16%
[40]		Multiclass classification MobileNet, DenseNet201, InceptionNetV2 and NasNetMobile	MobileNet Accuracy: 91.26%
			DenseNet201Accuracy: 90.38%
			InceptionNetV2 Accuracy: 89.27%
			NasNetMobile Accuracy: 87.74%
**Proposed method**		**RVCNet**	**Accuracy 91.27%, AUC 92.31%, precision 90.48%, recall 98.30%, f1-score 94.23% and loss 27.85%.**

## 8. Conclusion

This paper proposes a new DL framework called RVCNet, which effectively integrates ideas from ResNet101V2, VGG19, and basic CNN concepts. By leveraging stacking ensemble and concatenation techniques, as well as hyperparameter fine-tuning and additional layers, the model aims to achieve improved classification accuracy. The new model was developed for four types of lung disease categorizations, comparing X-ray images of healthy people with patients with COVID-19, non-COVID lung infections, and viral pneumonia. The proposed RVCNet demonstrated a reliable classification accuracy of 91.27% when classifying the four classes of COVID-19, viral pneumonia, non-COVID pneumonia, and normal patients. Additionally, the AUC, precision, and recall of the model were 92.31%, 90.48%, and 98.30%, respectively. Finally, the model identifies lung diseases and leverages GRAD-CAM heatmaps on test images to decode classification outcomes, underlining essential image areas that impact classification and identifying vital features for disease distinction.

Despite the model’s ability to identify lung diseases using X-ray samples, its effectiveness depends on the dataset. The disease prediction may not be accurate if the dataset contains many noisy and distorted images. To increase correct predictions, any DL model must be trained using a large dataset. As a future direction, the effectiveness of the proposed model could be assessed for infectious diseases like pneumonia, COVID-19, or other viral and bacterial infections in the context of larger datasets consisting of samples from diverse populations, and more than four class classifications. Additionally, a comparative study could be conducted to investigate the performance of the stacking ensemble against other ensemble methods, such as voting, bagging, and boosting, to provide further justification for the preference of the stacking ensemble in this study. Finally, continuous research is needed to build more accurate, reliable, and practically useful DL-based lung disease detection systems, ultimately improving patient care and outcomes in respiratory medicine.
